# Worsening cholestasis and possible cefuroxime-induced liver injury following “successful” therapeutic endoscopic retrograde cholangiopancreatography for a distal common bile duct stone: a case report

**DOI:** 10.1186/s13256-016-1123-0

**Published:** 2016-12-21

**Authors:** Madunil Anuk Niriella, Ravindu Sujeewa Kumarasena, Anuradha Supun Dassanayake, Aloka Pathirana, Janaki de Silva Hewavisenthi, Hithanadura Janaka de Silva

**Affiliations:** 1 Faculty of Medicine, University of Kelaniya, Ragama, Sri Lanka; 2 Colombo North Teaching Hospital, Ragama, Sri Lanka; 3 Faculty of Medical Sciences, University of Sri Jayawardenapura, Colombo, Sri Lanka

**Keywords:** Cefuroxime, Drug-induced liver injury, Cholestasis, ERCP

## Abstract

**Background:**

Cefuroxime very rarely causes drug-induced liver injury. We present a case of a patient with paradoxical worsening of jaundice caused by cefuroxime-induced cholestasis following therapeutic endoscopic retrograde cholangiopancreatography for a distal common bile duct stone.

**Case presentation:**

A 51-year-old, previously healthy Sri Lankan man presented to our hospital with obstructive jaundice caused by a distal common bile duct stone. Endoscopic retrograde cholangiopancreatography with stone extraction, common bile duct clearance, and stenting failed to improve the cholestasis, with paradoxical worsening of his jaundice. A liver biopsy revealed features of drug-induced intrahepatic cholestasis. Although his case was complicated by an episode of cholangitis, the patient made a complete recovery in 4 months with supportive treatment and withdrawal of the offending drug.

**Conclusions:**

This case highlights a very rare drug-induced liver injury caused by cefuroxime as well as our approach to treating a patient with paradoxical worsening of jaundice after therapeutic endoscopic retrograde cholangiopancreatography.

## Background

Cefuroxime is a second-generation cephalosporin known to very rarely cause drug-induced liver injury (DILI). There have been fewer than five previously reported cases of cefuroxime causing DILI and cholestasis. In a follow-up of a previous report of liver injury caused by ampicillin, Köklü *et al*. [1] described a case of a 23-year-old man who redeveloped liver injury 17 days after starting a 10-day course of oral cefuroxime (bilirubin 0.7 mg/dl, alanine transaminase [ALT] 427 U/L, alkaline phosphatase [ALP] 646 U/L), which resolved within 2 months, suggesting cross-reactivity with ampicillin. In a report by Chalasani *et al*. for the Drug-Induced Liver Injury Network [2], among 300 cases of DILI in the United States collected between 2004 and 2008, 5 cases were attributed to cephalosporin, with single cases linked to cefuroxime. In another report, Ekiz *et al*. described a 60-year-old woman who developed jaundice 4 days after a 10-day course of oral cefuroxime (bilirubin 17.9 mg/dl rising to 30 mg/dl, ALT 1527 U/L, ALP 1006 U/L), with progressive worsening of international normalized ratio (INR) [1.9] and referral for transplant but with a subsequent full recovery [3]. Given the paucity of case reports of cefuroxime-induced DILI, this drug belongs to category D (one to four reported cases) according to a recently suggested categorization of drugs implicated in hepatotoxicity and DILI [4]. In this report, we present a case of a patient with paradoxical worsening of jaundice due to possible drug-induced cholestasis caused by cefuroxime following therapeutic endoscopic retrograde cholangiopancreatography (ERCP) for distal common bile duct (CBD) obstruction by a stone.

## Case presentation

A previously healthy, 51-year-old Sri Lankan man presented with right upper quadrant colicky abdominal pain of 3 days’ duration. The pain was associated with yellow discoloration of the eyes, passage of dark urine, and generalized itching. He had no significant past medical, environmental, or social history. His clinical examination revealed that he was afebrile but had deep icterus, excoriations, and mild right upper quadrant abdominal tenderness. He had no stigmata of chronic liver disease.

The results of laboratory investigations included a normal full blood count and inflammatory markers, deranged liver biochemistry (total bilirubin 6.4 mg/dl, ALP 325 IU/L, aspartate transaminase [AST] 113 U/L, ALT 318 U/L), normal liver function (serum albumin 3.8 g/dl, serum globulin 2.6 g/dl, INR 1.00, activated partial thromboplastin time [APTT] 29 seconds), and normal renal profile. An ultrasound scan (USS) of the abdomen showed the presence of cholelithiasis with features of chronic cholecystitis and a dilated CBD and intrahepatic ducts due to a distal CBD obstruction. Contrast-enhanced computed tomography of the abdomen confirmed the presence of a distal CBD stone causing proximal CBD and intrahepatic duct dilation and cholelithiasis.

The patient underwent ERCP with sphincterotomy and balloon extraction of a CBD stone 2 weeks from the onset of symptoms. After surgery, the patient was given intravenous cefuroxime 750 mg three times daily for 1 day, followed by oral cefuroxime 500 mg twice daily for 5 days. The patient’s symptoms and biochemistry failed to improve, with worsening of cholestasis (total bilirubin 20.3 mg/dl, ALP 537 IU/L) following the “successful” therapeutic ERCP. Repeat ERCP 1 week later showed a normal CBD with no residual CBD stones. A 10-French, 10-cm CBD stent was inserted at this stage.

The patient was then referred to a hepatologist for evaluation of worsening jaundice post-ERCP. By this time, the patient’s obstructive jaundice symptoms were severe and disabling, and biochemical analysis revealed worsening cholestasis (total bilirubin 39 mg/dl, ALP 651 IU/L), relatively normal liver enzymes (AST 61 U/L, ALT 62 U/L, gamma-glutamyltransferase [GGT] 25 U/L] with normal liver function (serum albumin 3.7 g/dl, serum globulin 1.9 g/dl, INR 1.00, APTT 29 seconds), normal full blood count, normal inflammatory markers, and normal renal profile. Repeat USS of the abdomen revealed multiple cholelithiasis, chronic cholecystitis, stent in the CBD, and no intrahepatic biliary radical dilation. Test results for the patient’s hepatitis A immunoglobulin M (IgM), hepatitis E antibodies, hepatitis B surface antigen, anti-hepatitis C antibodies, and *Leptospira* IgM antibodies were negative.

**Fig. 1:**
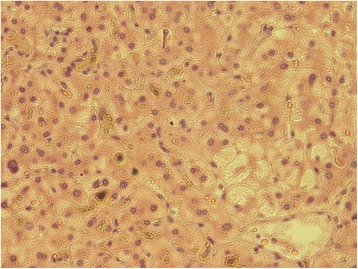
Hepatic parenchyma around the central vein (*lower left*) showing cholestasis, feathery degeneration, inflammation, and lobular disarray (hematoxylin and stain, original magnification ×400)

On the basis of all the above-mentioned information, an ultrasound-guided liver biopsy was performed. The patient was commenced on prednisolone 40 mg daily, ursodeoxycholic acid 300 mg three times daily, and cholestyramine 5 g three times daily for symptomatic relief. A liver biopsy showed no evidence of portal tract edema or inflammation, lesions or paucity of bile ducts, bile infarcts or leaks, or inflammation or interphase hepatitis. However, there was marked bilirubinostasis in zone 3 canaliculi, with surrounding hepatocytes revealing feathery degeneration and surrounding inflammation (Fig. 1). These features were compatible with intrahepatic cholestasis related to either drugs or sepsis.

One week later, the patient developed fever with chills and rigors, worsening cholestasis clinically and biochemically (total bilirubin 48 mg/dl, ALP 901 IU/L) with relatively normal liver enzymes (AST 55 U/L, ALT 91 U/L, GGT 43 U/L), raised inflammatory markers (C-reactive protein 36 mg/dl), and neutrophil leukocytosis (white blood cell count 14,400/mm^3^ with 80% neutrophils). A clinical diagnosis of cholangitis was made, and the patient was commenced on intravenous meropenem 1 g three times daily, after blood cultures, for 14 days. The CBD stent was removed endoscopically to exclude the possibility of a blocked stent as the precipitant of cholangitis. Oral prednisolone was tapered rapidly in view of the infection. The cholangitis episode settled with the antibiotics, with resolution of fever and normalizing of full blood count and inflammatory markers.

The patient’s cholestasis improved gradually over the next 2 months with ursodeoxycholic acid therapy. He had complete resolution of both clinical and biochemical features (total bilirubin 1.1 mg/dl, ALP 135 IU/L) of cholestasis 4 months into his illness. He was referred back to a hepatobiliary surgeon for elective laparoscopic cholecystectomy for residual cholelithiasis to prevent recurrence of complications and was advised to avoid cephalosporin use in the future. The timeline of the patient’s clinical course is presented in Table 1.

**Table 1:** Timeline of the patient’s clinical course

Time	Total bilirubin (mg/dl)	ALP (IU/L)	AST (IU/L)	ALT (IU/L)	GGT (IU/L)	Albumin (g/dl)	Globulin (g/dl)	FBC	CRP (mg/dl)
Initial presentation with right upper quadrant pain, jaundice, dark urine, and pruritus									
Day 3	6.4	325	113	318		3.8	2.6	Normal	<6
The patient underwent ERCP on day 14, followed by cefuroxime for 5 days.									
Day 17	20.3	537	29	47		4.1		Normal	<6
The patient underwent repeat ERCP on day 21 (1 week after the therapeutic ERCP), and a CBD stent was inserted. He was then referred to a hepatologist for worsening obstructive jaundice.									
Day 23	32.8	364	42	40		3.6		Normal	<6
The patient was commenced on prednisolone 40 mg daily, ursodeoxycholic acid 300 mg, and cholestyramine 5 g three times daily. The patient was readmitted with fever with rigors and worsening of jaundice on day 32.									
Day 32	39	651	62	61	25	3.7	1.9		
Stent removed endoscopically on day 33. The patient was treated for cholangitis with intravenous meropenem for the next 14 days; prednisolone was tapered; ursodeoxycholic acid 300 mg and cholestyramine 5 g three times daily were continued.									
Day 34	35	595	57	52	18	3.23	1.57	Neutrophil leukocytosis (white blood cell count 14,400/mm^3^ with 80% neutrophils)	42
Day 36	48	901	55	91					36
Day 40	38	752							
4 months after onset of illness	1.1	135	30	40					

*Abbreviations: ALP* Alkaline phosphatase, *ALT* Alanine transaminase, *AST* Aspartate transaminase, *CBD* Common bile duct; *ERCP* Endoscopic retrograde cholangiopancreatography *GGT* Gamma-glutamyltransaminase, *FBC* Full blood count, *CRP* C-reactive protein

## Discussion

This case is unique and complicated, involving a very rare DILI secondary to cefuroxime in the setting of therapeutic ERCP for distal CBD obstruction. Previously reported cases of DILI caused by cefuroxime were not complicated by preexisting obstructive jaundice.

The cholestasis in our patent, which was due to distal CBD obstruction by a stone, was expected to improve following therapeutic ERCP. However, despite successful stone extraction, CBD clearance, and stenting, our patient’s cholestasis paradoxically continued to worsen.

Worsening cholestasis post-ERCP may be due to a retained CBD stone, ascending cholangitis, a blocked or migrated stent, or drug-induced cholestasis. In our patient, the most likely reason was drug-induced cholestasis. This was suggested by the absence of features of sepsis following initial ERCP, the absence of ductal dilation seen on an USS with stent in situ in the CBD, and the features present on liver histology favoring intrahepatic cholestasis of drug-induced etiology. The agents that may be responsible for this may be the drugs used during or after ERCP. Our patient was given propofol, pethidine during ERCP, and cefuroxime post-ERCP. All of these agents are known to cause cholestasis, but cefuroxime seems the likely causative agent.

Cefuroxime is a second-generation cephalosporin very rarely known to cause DILI. Prior to the present report, fewer than five cases of cefuroxime causing DILI and cholestasis had been reported [1–3]. Although the exact mechanism of cholestatic DILI of cefuroxime is not known, it is likely idiosyncratic in nature and due to hypersensitivity. Conversely, a closely related third-generation cephalosporin, ceftriaxone, is known to cause biliary sludge formation because of the propensity of ceftriaxone to bind calcium and form insoluble crystals in bile in the gallbladder, resulting in biliary sludge or frank stones [5].

Our patient’s cholestasis worsened further following an episode of cholangitis, which was successfully treated with meropenem, thereby avoiding further exposure to cephalosporin. Following successful treatment of his cholangitis, the patient was managed with only ursodeoxycholic acid and cholestyramine. This resulted in gradual resolution of his cholestasis, with complete resolution in 4 months.

The place of steroids in drug-induced cholestasis is equivocal [6]. Corticosteroids can be effective for DILI associated with autoimmune or systemic hypersensitivity features. In our patient, there was no marked improvement of cholestasis on commencing steroids. Furthermore, the episode of cholangitis was worsened by steroids. Therefore, steroids were tapered rapidly at the onset of cholangitis with antibiotic cover.

## Conclusions

Our patient’s case illustrates a rare DILI due to cefuroxime, presenting as prolonged cholestatic jaundice following “successful” therapeutic ERCP for an obstructing distal CBD stone. Clinicians must be aware of the causes of worsening cholestasis post-ERCP to manage as well as to prevent the recurrence of similar complications in such patients.

## Abbreviations

ALP: Alkaline phosphatase
ALT: Alanine transaminase
APTT: Activated partial thromboplastin time
AST: Aspartate transaminase
CBD: Common bile duct
CRP: C-reactive protein
DILI: Drug-induced liver injury
ERCP: Endoscopic retrograde cholangiopancreatography
FBC: Full blood count
GGT: Gamma-glutamyltransferase
IgM: Immunoglobulin M
INR: International normalized ratio
USS: Ultrasound scan
